# Scrutinizing ciliopathies by unravelling the ciliary interactome

**DOI:** 10.1186/2046-2530-1-S1-P58

**Published:** 2012-11-16

**Authors:** J van Reeuwijk, K Boldt, DA Mans, Y Texier, SE van Beersum, SJ Letteboer, MTT Nguyen, M Ueffing, R Roepman

**Affiliations:** 1 Department of Human Genetics, Nijmegen Centre for Molecular Life Sciences, Radboud University Nijmegen Medical Centre, the Netherlands; 2 Medical Proteome Center, Center of Ophthalmology, University of Tuebingen, Germany; 3 Department of Protein Science, Helmholtz Zentrum München, German Research Center for Environmental Health, Neuherberg, Germany; 4 Institute for Genetic and Metabolic Disease, Radboud University Nijmegen Medical Centre, the Netherlands

## 

Recent (affinity) proteomics studies have focused on the composition and dynamics of ciliary protein interaction networks. This has unveiled important knowledge about the highly ordered, interconnected but very dynamic nature of the cilium as a molecular machine in the cell. Disruption of members of functional modules of this machine leads to overlapping phenotypes. Detailed analyses of the binding repertoire, the biochemical properties and the biological functions of such modules have yielded the identification of new ciliopathy genes as well as new insights into the pathogenic mechanisms underlying ciliopathies. To gain better insights into the molecular disease mechanisms that underlie ciliopathies and to acquire knowledge about the general importance of the cilium for cellular homeostasis, we are conducting ciliary interactome studies and will combine this information with subcellular localization data, and functional data derived from gene knockdown in ciliated cells or knockout/mutant vertebrate models. This integrated dataset enables us to generate models of interacting functional modules associated with cell signalling cascades, developmental events or specific ciliary functions such as intraflagellar transport. To date, we have performed affinity proteomics for the majority of known ciliopathy-associated proteins and many other ciliary proteins. In addition, yeast two-hybrid experiments have been performed to study the physical interaction between ciliary proteins in more detail. Our current dataset shows high interconnectivity between many of the ciliopathy-associated proteins. These may be part of functional ciliary modules as many are associated with clinically overlapping phenotypes. In addition, new members of these modules are excellent novel candidate ciliopathy proteins.

